# Apathy in Frontotemporal Dementia: Behavioral and Neuroimaging Correlates

**DOI:** 10.3233/BEN-2011-0351

**Published:** 2012-01-20

**Authors:** Paul J. Eslinger, Peachie Moore, Shweta Antani, Chivon Anderson, Murray Grossman

**Affiliations:** ^1^Departments of NeurologyNeural and Behavioral Sciencesand Radiology College of MedicinePenn State Hershey Medical CenterHersheyPAUSA; ^2^Department of NeurologyUniversity of Pennsylvania School of MedicinePhiladelphiaPAUSA

**Keywords:** Frontotemporal dementia, apathy, social cognition, executive function, frontal, parietal, caudate

## Abstract

We investigated the occurrence of goal-directed motivational change in the form of apathy in patients with frontotemporal dementia (FTD), particularly those with behavioral variant social and executive deficits (bvFTD). Standardized behavioral inventory was employed to survey and compare apathy ratings from patients and caregivers. In cases of bvFTD, apathy ratings were further related to measures of social cognition, executive function, and atrophy on brain MRI. Results indicated that caregivers rated bvFTD patients as having significantly elevated apathy scores though patient self-ratings were normal. Caregiver and self-ratings of FTD samples with progressive nonfluent aphasia and semantic dementia did not differ from healthy controls and their informants. In the bvFTD sample, caregiver apathy scores were not correlated with general cognitive screening or depression scores, but were significantly correlated with social cognition and executive function measures. Voxel-based morphometry revealed that apathy ratings in bvFTD were related to prominent atrophy in the right caudate (including the ventral striatum), the right temporo-parietal junction, right posterior inferior and middle temporal gyri, and left frontal operculum- anterior insula region. Findings suggest that bvFTD is associated with a significant breakdown in goal-directed motivated behavior involving disruption of cortical-basal ganglia circuits that is also related to social and executive function deficits.

## 1. Introduction

Recent investigations have begun to identify underlying deficits and correlates of the clinically problematic social and executive function impairments in frontotemporal dementia, particularly in those patients who present with significant behavioral deficits [i.e., behavioral variant FTD (bvFTD) [13,30,42,44]]. Initial symptoms can be subtle and perplexing, as these patients do not show striking memory, language, sensorimotor or spatial deficits, but rather symptoms that can be interpreted as depression, adjustment problems, stress, or lapses of judgment and self-control. One of the more enigmatic features concerns insidious decline in motivation and typical goal-directed actions. As these behavioral symptoms progress, flattened, blunted, and poorly-regulated affect can be readily observed along with decreasing interest in usual social, recreational, occupational and creative pursuits, usually described as clinical apathy [25,38]. Although symptoms of clinical apathy have been reported to be more frequent in FTD than in Alzheimer's disease (AD) [7,15, 27], few studies have undertaken integrated analysis of its occurrence, neuropsychological correlates, and pathophysiology. Within the FTD clinical spectrum, apathy and related symptoms appear to be most apparent and problematic in bvFTD cases (15, 35, 51), which is the focus of this investigation.

The construct of apathy refers to the pervasive decline in typical motivated behaviors that is not otherwise attributable to altered consciousness, cognition, or emotional distress. Investigators have suggested that as a clinical characteristic of FTD, particularly in bvFTD

ISSN 0953-4180/12/$27.50 © 2012 – IOS Press and the authors. All rights reserved samples, apathy can be differentiated from depression and associated dysphoric symptoms, and hence may arise from a different neural substrate and pathological process [27], although this hypothesis has not been examined in detail. Clinical presentations of apathy have been linked to atrophy of the frontal lobes (including dorsolateral, anterior cingulate, orbital and medial regions) and to right frontal hypoperfusion [30,35,36,44, 51]. Apathy has also been associated with greater functional impairments and caregiver distress. However, no studies have specifically examined the relationship between apathy and neuropsychological impairments in FTD. In the case of bvFTD, such measures would need to encompass the major domains of impairment including social cognition and executive function. Levy et al. [27] reported that apathy was correlated with a measure of general cognition (Mini Mental State Exam) in FTD. AD studies have found that patients with apathy score significantly worse than those without apathy on executive function measures such as Trail making, Stroop, set shifting and associative fluency [26, 37]. Similar analyses in FTD would be important because of the suspected role of apathy in the behavioral changes affecting social comportment and independent functioning.

In this study, we sought to survey the characteristics of apathy in FTD, including bvFTD, semantic dementia (SD), and progressive nonfluent aphasia (PNFA) samples, using a reliable and well-validated instrument (the Apathy Evaluation Scale [34]) and garnering the observations and ratings of both patients and caregivers. Based on prior clinical studies, we suspected that apathy would be identified more frequently in the bvFTD sample and more so by caregivers than patients. If this supposition is confirmed, we planned to examine apathy data in relationship to measures of social cognition and executive functions which are the major domains of clinical impairment in bvFTD and where significant correlations might reasonably be expected. Such analyses have not been reported to date. Further, we suspected that apathy would be related to specific neurodegenerative changes in the brain, and in particular to frontal and/or basal ganglia regions. Studies relating apathy to cortical atrophy in bvFTD samples have emphasized reduction of tissue volume in prefrontal cortex (including rostral, anterior cingulate, dorsolateral, and lateral orbitofrontal regions) and striatum [35,44,51], although agreement on striatal involvement is uncertain [29]. In this study, we planned to examine the neural substrate for apathy in bvFTD patients with voxel-based morphometry and hypothesized that significant change would be detected in rostral-medial structures that could include the basal ganglia, cingulate and/or frontal cortex.

In prior studies, bvFTD patients have been reported by caregivers to develop apathy-related changes to a greater extent than SD, PNFA and AD sample. bvFTD patients also suffer deficits in social cognition (including theory of mind, empathy and social problem solving) and executive processing resources such as cognitive flexibility and metacognition [15–17], although it remains unclear if these are associated with apathy. Recent family member survey from our lab identified that apathy is an important additional contributor to bvFTD patients' clinical impairment profile and associated with frontal disease [35]. Therefore, we sought to undertake a more integrative and quantitative analysis of the possible cognitive and neural associations of apathy in FTD.

## 2. Methods

### 2.1. Subjects

Twenty-six patients were recruited for studies directly by clinical investigators in the Neurology clinics of the Hospital of the University of Pennsylvania. Patients were diagnosed according to modifications of published criteria for FTD [11,32,33,40] with other dementia causes excluded by clinical exam, blood and brain imaging tests. Informed consent was provided by participants and caregivers according to University of Pennsylvania Institutional Review Board approved protocol.

Two independent examiners established consensus diagnoses for FTD as well as FTD subgroups as follows: bvFTD ($n = 12$), PNFA ($n = 7$) and SD ($n = 7$) patients. Participants were alert and cooperative, did not exhibit a motor disorder, and none of the patients were taking medications that interfere with attentiveness (e.g., benzodiazepines). Normal control (NC) participants ($n = 16$) were chosen to match FTD samples for age, education, and gender (see Table 1 for summary), differing only by higher Mini-Mental State Exam scores from the FTD samples ($p < 0.0001$) and marginally higher age than the bvFTD sample ($p < 0.05$).

Table 1
Summary characteristics (mean and standard deviation) of frontotemporal dementia sample and controls[a]

| Study sample | bvFTD | PNFA | SD | Control |
|---|---|---|---|---|
| Sample size | 12 | 7 | 7 | 16 |
| Age (years) | 65.3 (12.6) | 73.6 (7.3) | 68.7 (7.7) | 75.0 (6.6) |
| Education (years) | 14.8 (4.7) | 15.4 (2.7) | 13.9 (2.5) | 15.1 (2.5) |
| MMSE (max = 30) | 23.3 (5.4) | 21.7 (9.6) | 23.8 (5.8) | 29.3 (0.9) |

[a]bvFTD: Behavioral variant FTD; PNFA: Progressive Nonfluent Aphasia; SD: Semantic Dementia.

## 2.2. Behavioral and cognitive measures

### 2.2.1. Apathy

Symptoms of apathy were surveyed with the Apathy Evaluation Scale (AES [34]). Patients and their caregivers (as well as controls and their spouses or other informants) completed the 18-item rating scale that yielded an overall score as well as cognitive, behavioral, and insight subcomponent scores for goal-directed behavior. Sample items in the self-rating version included: "I am interested in having new experiences" and "I spend time doing things that interest me." Sample items in the caregiver/informant version included: "He/She gets things done during the day" and "He/She is interested in learning new things." The items in self- and caregiver/informant versions were identical except for the agency differences. Each item was rated on a 4-point Likert scale (4-Not at all true to 1-Very True), with reverse scoring required for items 6, 10 ardll. Total score ranged from 18–72. Hence, higher scores indicated more symptoms and behavioral indications of apathy. The AES has been reported to have satisfactory reliability and validity characteristics, including test-retest and inter-rater reliability as well as convergent and discriminant validity [see [8,34] for more details].

### 2.2.2. Depression

Survey of depressive symptoms was undertaken with the Beck Depression Inventory, a 22 item rating scale sensitive to severity of somatic-vegetative and cognitive-emotional symptoms of depression [3].

### 2.2.3. General cognition

General cognitive level was assessed with the Mini-Mental State examination [19].

Given the multidimensional character of executive functions, we included several standardized measures that have previously been shown to be sensitive to the executive function deficits in bvFTD, as follows.

### 2.2.4. Visual Verbal Test

This nonsocial measure of cognitive flexibility and executive resources required abstraction and response shifting. Stimulus sets were 4 geometric designs on a single page [18,49]. Participants categorized three of the designs in two different ways based on an abstracted similarity in color, shape, size or orientation (e.g., 3 designs having straight edges, another 3 designs having interior horizontal lines). The 10 stimulus sets were preceded by a practice item. First choice abstraction accuracy ranged 0–10, with score reported as percentage correct. Second choice abstraction accuracy provided a measure of set-shifting ability and was computed relative to first choice abstraction score and reported as percentage correct.

### 2.2.5. Trail Making Part B

Standard version of this number-letter sequencing and switching task as a measure of cognitive flexibility was completed, with scoring of amount of time to completion, up to 300 seconds [43].

### 2.2.6. Stroop Interference Test

Subjects identified the font color in 5 columns of 16 color names printed in a colored font that differed from the word (e.g., the word RED printed in a green font; correct response green), with scoring of amount of time to completion, up to 300 seconds [48].

Participants were also administered several measures of social cognition that have been shown to be sensitive to bvFTD, as follows.

### 2.2.7. Cartoon predictions

Based on an initial picture depicting a social dilemma, participants chose the next most likely action to address the dilemma from among three choices [41]. This measure assessed prediction of social consequences and was shortened to a 10-item version from the original Form A. No verbal responses were required. Performance was untimed after a practice item established that participants understood the task, and test scores are reported as percentage correct responses. FTD patients have previously been shown to be impaired on this task, particularly those with bvFTD [16].

*2.2.8. Theory of mind*

Twelve vignettes assessed judgment of story facts, first order and second order beliefs of characters in social situations [50]. Each scenario was presented with a *lie* and *joke* condition that required contrasting decisions about a main character's first order beliefs, and the second order beliefs that another character held of the main character. Second order belief questions also required interpretation of whether the character was lying to avoid getting caught or joking to cover up embarrassment. Hence, a total of 24 vignettes were presented with 3 theory of mind responses to each vignette (first order belief, second order belief, and interpretation of second order beliefs). Results are reported as percentage correct for each type of response. Vignettes were read aloud and concurrently presented in written format. A practice vignette was presented to establish that participants understood the task. Comprehension of the task was also established by analysis of first order beliefs which were not statistically different between bvFTD and control samples. bvFTD patients have been shown to be particularly impaired with second order belief processing [16,31].

*2.2.9. Empathy scale*

Patients and their caregivers (as well as controls and their spouses or other informant) completed the Interpersonal Reactivity Index [10], a reliable and valid 28-item inventory of empathy that yields a total score as well as 4 subscale scores (Perspective-Taking; Fantasy; Empathic Concern; and Personal Distress). For these analyses, we focused on total scale score and more specifically on cognitive and emotional aspects of empathy by contrasting the Perspective-Taking and Empathic Concern subscales. Sample items for the Perspective-taking subscale included: "I try to look at everybody's side of a disagreement before I make a decision" and "Before criticizing somebody, I try to imagine how I would feel if I were in their place." Sample items for the Empathic Concern subscale included: "When I see someone being taken advantage of, I feel kind of protective toward them" and "I often have tender, concerned feelings for people less fortunate than me." Each item was rated on a 5-point Likert scale ranging from 0 (does not describe me well) to 4 (describes me very well). Total scale score ranged from 0–112, and subscale scores ranged from 0–28. Hence, higher scores indicated more empathic capacities, and lower scores indicated less. bvFTD patients are frequently rated by caregivers as being low in empathy capacities, while their self-ratings can lack awareness of any change and be normal [15,17].

Statistical analysis of the abovementioned dependent measures was undertaken with analysis of variance, post hoc Scheffe tests, and Pearson product-moment correlational analysis, with Bonferroni correction for significance noted.

*2.3. Imaging procedure*

High resolution structural MRI images were obtained for bvFTD patients in a Siemens Trio 3T MRI scanner. After rapid sagittal T1-weighted imaging to determine patient position, high resolution T1-weighted 3D MPRAGE images were acquired with repetition time (TR) of 1620 msec, echo time (TE) of 30 msec, 1 mm slice thickness, flip angle of 15 degrees, matrix size of 192 $\times$ 256, and rectangular field of view giving an in-plane resolution of 1.0 $\times$ 1.0 mm. Images were processed as follows. First, brain volumes were registered in SPM99 [21] using 12-parameter affine registration, nonlinear registration using 12 nonlinear iterations, and 7 $\times$ 8 $\times$ 7 basis functions. Brains were normalized to the T1 template of 305 averaged brain volumes with standardized brain coordinates using a high dimensional normalization procedure [22]. Brain volumes were segmented into four tissue types (gray matter, white matter, CSF, other). The segmentation algorithm in SPM99 calculates a Bayesian probability for each tissue type voxel in the volume, based on a priori MRI information. We inspected each slice of each segmented volume to ensure that no voxels from the dural sinuses or adjacent non-brain structures were misclassified as gray matter. Using SPM99, the gray matter volume was smoothed with a 12 mm FWHM Gaussian filter to minimize individual gyral variations.

Voxel-based morphometry (VBM) in SPM99 analyzed brain volumes [2]. A proportional analysis threshold included only voxels with 40% or more of the grand mean value. Implicit masking was used to ignore zeros, and global calculation was based on the mean voxel value. SPM99 analyses included a two sample t-test routine to compare the gray matter volume of bvFTD patients to 12 healthy, age-matched controls. Statistical threshold for atrophy studies relative to controls was set at $p < 0.0001$. We did not correct for multiple comparisons because of the hypothesis-driven nature of the analyses. Moreover, the small size of the voxels would make Bonferroni-like statistical correction too conservative. Instead, only clusters of 100 or more adjacent voxels were considered, reflecting a statistically

Table 2
Apathy Evaluation Scale (AES) scores for FTD clinical subgroups and the healthy control sample

| AES scores | Cognitive | Behavioral | Insight | Total[a] |
|---|---|---|---|---|
| *Controls* | | | | |
| Self rating | 15.54 | 8.46 | 2.15 | 35.15 |
| Informant rating | 14.22 | 7.44 | 1.56 | 30.44 |
| Discrepancy | −1.10 | −1.00 | −0.50 | −3.70 |
| *FTD Samples* | | | | |
| *Behavioral variant* | | | | |
| Self rating | 16.54 | 9.54 | 1.36* | 34.36 |
| Informant rating | 26.50* | 14.42* | 3.50* | 58.25* |
| Discrepancy | 9.60* | 4.70* | 2.20* | 23.20* |
| *Progressive Nonfluent Aphasia* | | | | |
| Self rating | 17 | 8.20 | 2.00 | 35.60 |
| Informant rating | 17 | 9.00 | 2.20 | 38.40 |
| Discrepancy | 0 | 0.57 | 0.14 | 2.00 |
| *Semantic Dementia* | | | | |
| Self-rating | 13.11 | 8.67 | 2.33 | 30.44 |
| Informant rating | 16.33 | 10.00 | 2.33* | 38.11 |
| Discrepancy | 3.71 | 2.00 | −0.14 | 8.14 |

[a]Not all AES items fit into a subscale; hence total AES score is greater than the sum of subscales.
*Significant difference from Controls ($p < 0.001$).

robust effect exceeding $p < 0.05$ corrected for multiple comparisons in this neuroanatomic distribution [20]. Regression analysis relating AES scores to gray matter atrophy derived from contrast of cortical atrophy in the bvFTD group relative to control subjects. Statistical threshold for these analyses was set at $p < 0.025$ uncorrected, requiring $> 100$ adjacent voxels/cluster.

## 3. Results

### 3.1. Apathy scores

Self-ratings of apathy-related behaviors on the AES did not differ among FTD patient groups and controls (See Table 2 for summary scores). In particular, the bvFTD sample did not acknowledge any change in their goal-directed and motivated behaviors. In contrast, AES caregiver ratings identified a marked increase in apathy scores for the bvFTD sample (AES total score = 58.25) which were significantly higher than the PNFA, SD, and healthy control samples (AES total scores = 30.44–38.40, $p < 0.0001$). The bvFTD sample was rated significantly higher in cognitive, behavioral and insight subscores of the AES in comparison to all other samples ($p < 0.001$). The PNFA and SD samples did not differ from controls or from each other.

#### 3.1.1. Discrepancy analysis

In comparison to controls and their informants, the bvFTD patients and respective caregivers differed markedly in their apathy appraisals. bvFTD patients viewed themselves as essentially the same as controls, whereas their caregiver ratings were significantly elevated on total score and all AES components, indicating more apathetic behaviors and symptoms ($p < 0.001$ for all contrasts). The marked disparity between higher caregiver and lower patient-ratings of apathy in the bvFTD sample is also consistent with both clinical descriptions and other inventory comparison studies. Hence, we utilized caregiver ratings of apathy for analysis with other study measures below.

### 3.2. Executive function and social cognition measures in bvFTD patients

Multiple measures of executive function and social cognition were impaired in the bvFTD sample (See Table 3 for summary). Caregiver AES scores failed to correlate with screening mental status score ($p = 0.85$) and with depression score ($p = 0.34$) in the bvFTD sample. However, caregiver AES ratings were significantly correlated with the executive function measures of total time to completion on Trails B ($r = 0.72$, $p < 0.05$), and inversely correlated with the social cognition measures of Theory of Mind second order interpretation accuracy ($r = -0.71$, $p < 0.03$), and caregiver-rated empathy scales (Total Empathy Scale score $r = -0.70$, $p = 0.012$; Empathic Perspective-Taking $r = -0.51$, $p < 0.05$; Empathic Concern $r = -0.57$, $p < 0.03$). These findings indicated that apathy changes observed

Table 3
Summary of executive function, social cognition and mood scores in the bvFTD and control samples

| | bvFTD | Controls | p Value |
|---|---|---|---|
| *Executive Functions* | | | |
| Visual Verbal Test-First Choice | 89.17 (19.75) | 100 (0.00) | 0.05 |
| Visual Verbal Test-Second Choice | 46.89 (27.97) | 92.67 (8.84) | $0.0001^+$ |
| Trail Making Part B | 232.88 (90.19) | 123.71 (60.78) | 0.018 |
| Stroop Interference Test | 218.43 (80.23) | 97.17 (34.99) | 0.006 |
| *Social Cognition* | | | |
| Theory of Mind-First Order Beliefs | 85.42 (15.27) | 98.62 (3.40) | 0.109 |
| Theory of Mind-Second Order Beliefs | 58.33 (22.71) | 83.33 (14.91) | 0.029 |
| Theory of Mind-Second Order Interpretation | 44.79 (11.73) | 80.56 (14.59) | $0.001^+$ |
| Cartoon Predictions | 45.46 (28.06) | 84.71 (17.36) | $0.0001^+$ |
| Empathy Scale Total Score | 36.83 (11.76) | 53.78 (10.92) | $0.0001^+$ |
| Empathy Scale Perspective-Taking Score | 6.83 (3.37) | 17.80 (3.80) | $0.0001^+$ |
| Empathy Scale Emotional Concern Score | 11.67 (6.00) | 17.67 (4.03) | $0.005^+$ |
| *Mood* | | | |
| Depression Inventory | 14.67 (7.39) | 6.4 (4.68) | 0.006 |

$^+$Bonferroni correction for multiple comparisons; threshold $p = 0.004$.

Table 4
Voxel-based morphometry results (masked by atrophy) in the bvFTD sample showing the areas of atrophy significantly related to Apathy Evaluation Scale ratings by caregivers

| Anatomic locus (Brodmann area) | Coordinates | | | Z-score | Cluster |
|---|---|---|---|---|---|
| | X | Y | Z | | |
| R. Caudate | 10 | 18 | −4 | 3.50 | 262 |
| R. Temporo-Parietal Junction (39, 40) | 56 | −36 | 28 | 3.07 | 100 |
| R. Inferior-Middle Temporal Gyri (21/37) | 68 | −34 | −10 | 3.26 | 215 |
| L. Frontal operculum-Insula (44, 45) | −38 | 4 | 14 | 3.50 | 117 |

in bvFTD patients significantly related to decline in executive functions as well as social cognition. Self-ratings of apathy in the bvFTD samples correlated only with Theory of Mind second order belief accuracy ($r = 0.839, p = 0.009$).

### 3.3. *Voxel based morphometry in bvFTD patients*

Cortical atrophy on MRI scan was statistically related to caregiver ratings of apathy for the bvFTD subgroup (Fig. 1 and Table 4). Several significant clusters were evident in the right hemisphere in the regions of the head of the caudate (including the ventral striatum), the posterior portions of the middle and inferior temporal gyri, and the right temporo-parietal junction. In the left hemisphere, a significant cluster in the frontal operculum and anterior insula was identified.

## 4. Discussion

Results support the hypothesis that an identifiable association exists between behavioral assessment of apathy in bvFTD patients and their social cognitive as well as executive function capabilities. Hence, the potential impact of apathy on social and cognitive functioning of these patients is an important clinical issue that deserves assessment and treatment considerations. Standardized caregiver ratings of apathy-related behavioral changes in FTD identified marked increases in apathy disproportionately among bvFTD patients. This finding is underscored by the fact that there were no evident changes detected in bvFTD patient self-ratings, nor in the caregiver and patient ratings of the PNFA and SD samples. The pattern of results is consistent with prior clinical observations [7,30,35,38] and now couples the marked increases in apathy identified in the bvFTD sample with significant implications for executive function and social cognitive capacities. That is, caregiver apathy ratings were found to be significantly correlated with executive function, theory of mind, and empathy impairments in the bvFTD sample, accounting for up to approximately 50% of the variance in several measures. These associations appear to be not only sensitive but also relatively specific in bvFTD as apathy did not correlate with the general cognitive screening score of the Mini Mental State exam nor the depression screening score. Hence, the identification of apathy appears to signal important implications for

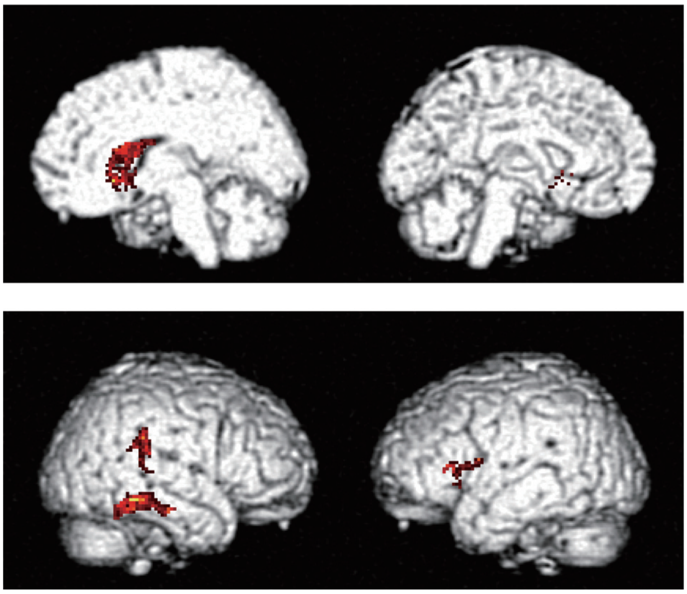

Fig. 1. Areas of atrophy that are significantly related to caregiver ratings of bvFTD patients on the Apathy Evaluation Scale.

patient management and caregiver resources beyond the detection of a dementia.

The results suggest parallel decline in motivational, executive function, and social domains in bvFTD that are commonly associated in a large-scale neural network that includes basal ganglia and prefrontal regions. Similar relationships between apathy and executive function measures have been reported in AD [26, 37] and hence the potential importance of apathy assessment and treatment in dementia is worth highlighting. Furthermore, current results extend the findings of Boone et al. [6] who reported that the increased *negative symptoms* detected in FTD were inversely correlated with executive functions and add the new findings that apathy is also inversely correlated with social cognitive measures of theory of mind and empathy in bvFTD cases. Moreover, apathy scores were dissociated from depression, supporting the important distinction of disordered motivation and goal-directed behavior in bvFTD from a disorder of mood.

Clarke et al. [8] emphasized the necessity of informant report for detection of apathy-related changes in FTD with the AES. Our comparisons of patient and caregiver behavioral ratings confirmed this observation and revealed a remarkable loss of self-awareness in bvFTD pertaining to apathy. The degree of caregiver-patient discrepancy was analyzed relative to healthy controls and their informants, insuring a conservative analysis that considered naturally occurring differences between participants and informants. The predominant pattern of the bvFTD patients was not to identify any apathy-related behavioral changes, in marked contrast to caregiver observations. This loss of personal awareness is consistent with systematic observations from other investigators and measures [8,15,45] as well as clinical correlation to right frontal and ventral pathology [35,44]. Thus, bvFTD patients exhibit not only significant social cognitive, emotional empathic, and motivational alterations, but also the loss of self-awareness of such dramatic changes, which is a key domain for adaptive social executive functions [14]. The specificity of these findings was underscored by the PNFA and SD samples that showed virtually no significant discrepancies from their informants.

In an anatomical analysis, we discovered that caregiver apathy scores of bvFTD patients were correlated most prominently with atrophic changes in the right basal ganglia, in particular the head of the caudate including the ventral striatum. This complex structure has been implicated in cognitive, affective and motor aspects of motivated behavior [1,5,9]. Progressive pathophysiology of this region, as posited by Levy and Dubois [28], significantly alters affective and cognitive aspects of motivated behavior which caregivers of the bvFTD patients confirmed in clear fashion. Moreover, the subsequent loss of *reward-related processing*, emotional responses to novelty, and usual interests suggests a marked deficiency in self-generated auto-activation as well in these patients. Links et al. [29] did not detect significant striatal change in FTD patients with apathy vs. no apathy, although the clinical characteristics of their FTD sample were unclear, there was no comparison to healthy controls, and the methods of MRI volumetric analysis were different. Our VBM analysis also identified that apathy was significantly correlated with atrophic changes in the right temporoparietal region. Extant data suggest that this region may mediate the integration of competing sources of situational data for purposes of contextualizing and scene-setting [12,46, 47]. In a mixed group of FTD patients, Zamboni et al. [51] similarly identified this region as being related to apathy measured via the Frontal Systems Behavior Scale.

In a neural systems model of social cognition, we have previously proposed that social and executive function domains can be integrated in a *social executor* framework of deficits in bvFTD patients [15], building upon studies in adult patients with acquired, focal lesions of the frontal lobe [14,23,24]. Specifically social executors encompass social knowledge and executive resources (including self-awareness, planning and self-regulation) as well as motivational and emotional components associated with interpersonal actions. Social knowledge elaborates the store of social perceptions, actions, experiences, and scripts that are bound by learned rules, conventions, and conditional probabilities, typically what is identified as social cognition. We suggest further that effective utilization of social knowledge is constrained by several allied processing resources including those that are specific to the social domain (e.g., theory of mind, empathic sensitivity), by domain-general executive resources (e.g., cognitive flexibility, self-awareness, self-regulation), and by goal-directed motivational and emotional influences that bias social perceptions and actions [4,39]. It is with respect to the latter processing resources that apathy may wield its effects on goal-directed motivation and emotions, subsequently impacting social cognitive and executive function capabilities

There are several limitations with respect to the study. The assessment and characteristics of apathy need to be assessed in a larger clinical sample and followed over the course of the disease for natural history and impact on morbidity/mortality. The survey and measurement of apathy also requires more of a systems-level analysis of symptoms, as suggested by Levy & Dubois [28]. This multidimensional analysis may lead to identification of anatomical regions beyond the basal ganglia that mediate important aspects of motivated and goal-directed behavior as well as components of apathy that may be amenable to different treatment strategies. The assessment of executive functions and social cognition within everyday contexts is also needed to validate the current measures and their relationship to apathy within everyday caregiving settings. Such analyses will help improve the generalizability and application of the findings to patient care. Finally, it is important to consider that in measures of depression among neurological patients, there may be different implications of somatic-vegetative and psychic-emotional symptoms as they relate to social and cognitive functioning as well as course of the disease.

The results support the conclusion that behavioral assessments of apathy in FTD, particularly based on caregiver observations, can reveal significant alterations in those patients who present with prominent social and executive impairments (i.e., bvFTD). Apathy was found to be significantly related to executive function and social cognition measures, suggesting that the behavioral impairments in bvFTD may emanate from convergence of multiple deficits associated with prefrontal cortex and related network systems. Caregiver ratings of apathy in bvFTD correlated significantly with atrophic changes in the region of the right caudate including the ventral striatum, suggesting further involvement of frontal-basal ganglia circuits as well as the right temporoparietal junction which has been implicated in social cognition, in the pathophysiology of apathy in FTD.

## Acknowledgement

This work was supported in part by the National Institutes of Health (AG17586, AG15116, NS44266, NS53488 and NS32953).
